# Residents’ future residential preference and its affecting factors in the rapid urbanization zone of rural China from a family life cycle perspective

**DOI:** 10.1038/s41598-024-64737-7

**Published:** 2024-06-14

**Authors:** Mengke Zhang, Yan Tong, Yuhang Ge, Jin Guo, Hanlin Nie, Zhijun Wang, Liangxin Fan

**Affiliations:** 1 School of Surveying and Land Information Engineering, Henan Polytechnic University, Jiaozuo, 454003 Henan China; 2 School of Architectural and Artistic Design, Henan Polytechnic University, Jiaozuo, 454000 China

**Keywords:** Residential preference, Homestead sites, Resident livelihood, Village planning, Rural China, Socioeconomic scenarios, Sustainability

## Abstract

Understanding farmers’ future residential preferences and the factors affecting these choices is crucial for tackling the issues related to hollow village management and rural planning. Despite limited research on the role of the family life cycle, this study explores how the family life cycle, characteristics of the household head, livelihood strategies, and resource availability shape farmers’ future residential preferences. Data were collected from 777 households in China’s main grain-producing area. The findings reveal that 52.90% of households prefer to stay in their current rural residences. Other favored options are elderly care facilities (13.90%), living with children in the village (12.36%), and ancestral homes (11.68%). The family life cycle significantly affects these preferences (*p* < 0.01), with changes in family structure and age leading to different living choices. Specifically, households in the initial (71.29%), burden (70.32%), and stable stages (40.14%) prefer their current rural residences, while those in the maintenance and empty-nest stages opt for living with their children’s residences (22.22% and 16.96%, respectively) or in elderly care facilities (30.00% and 33.93%). Meanwhile, age, health, income, livelihood strategies, and land ownership also markedly influence the choice of residence. Recommendations include educational programs for elderly rural residents, improving older individuals’ adaptability to rural changes, creating more rural employment opportunities, and enhancing medical and infrastructural services for the sustainable rural development.

## Introduction

Rapid urbanization significantly affects the relationship between urban and rural areas in developing countries, resulting in overcrowded cities, hollowed villages, and environmental degradation^1,2^. According to World Urbanization Prospects, the global population reached 8 billion in 2022, with the urban population projected to increase from 57% in 2022 to 68% by 2050, while the rural population is expected to decrease from 43% in 2022 to 32% by 2050^3^. The World Bank has reported a decline in rural populations in various regions. For example, rural India (1993–2022) witnessed a decline from 892 to 682 million, while urban populations rose from 242 to 460 million. In Africa, rural population (1993–2018) decreased from 886 to 789 million, with the urban populations increasing from 293 to 604 million. Similarly, rural China (1993–2022) experienced a decrease from 835 to 569 million.

In low-income countries, underdeveloped infrastructure, poor housing conditions^4^ and poverty^5,6^ are main barriers for rural development. In Asian countries, the imbalance in rural–urban development has led to the temporary internal migration of rural residents^7^. A significant number of rural residents have moved to cities and markedly developed regions in search of employment opportunities. Farmers’ education and their income involved in agriculture are the primary factors influencing their migration out of rural areas^8,9^. A survey of Bangladesh showed that economic gaps of rural–urban caused their seasonal migration, and household income and land ownership of farmers were key factors for their choice of future places of residence^10^.

China, being one of the most populous agricultural countries in the world^11^, places great importance on the development of rural areas for the overall economic and social stability of the nation. However, due to regional differences and imbalances, the process of urbanization in China’s rural areas involves substantial population movements, with farmers moving from rural areas to cities in pursuit of employment opportunities^12^. This kind of population mobility has given rise to various social problems, such as the issue of left-behind elderly and children, as well as rural hollowing. Understanding the preferences and needs of farmers regarding future residence, as well as the factors influencing these preferences and needs, is crucial for effective urban and rural planning^13^ and developing policies strategies for the Chinese rural development.

Numerous factors, including geographical location, environmental conditions, socio-economic background and infrastructure, influence farmers’ living preferences and future residence. In Yucatán Peninsula, Mexico, the distance to the city centre is main factor affecting farmers’ agricultural activities and living choices^14^. In Morocco, residents’ satisfaction with their current housing construction types relatively affects their future living choices^15^. Daniel et al.^16^ noted that Indonesia has a significant young population in rural areas, with many enterprises operating in these regions. Consequently, young Indonesians prefer staying in their current rural homes for the foreseeable future. In European countries, elderly households typically prefer rural living owing to their economic circumstances and lifestyle preferences^17,18^. In China, the migration of young people is considered to be a process closely related to economic factors. Family structure and income may affect their future living choices^19^. Chinese rural planning through rational allocation of rural construction land, thus improving farmers’ living satisfaction^20^. Among them, social networks and resource endowments also may affect farmers’ living choices by changing living environment in rural China.

The concept of the family life cycle, proposed by Rowntree^21^ and further developed by Glick^22^, provides a framework for understanding family decision-making and behaviour analysis involved in different families’ life cycle stages. Rowntree’s^23^ five-stage hypothesis, adjusted based on the characteristics of Chinese family social structure, includes young couple families, growing nuclear families, mature nuclear families, extended families, and empty nest couple families. Understanding the family life cycle is closely related to consumption, particularly in terms of how it affects expenditures across different consumption items and influences consumption intentions and decisions^24^. Newly married families exhibit the highest willingness to purchase housing, and the single stage is characterized by a vibrant rental market^25^. Farmers, from the standpoint of the family life cycle, have varying consumption structures and migration patterns.

The family life cycle leads to variations in family size, structure, and subsequently affects housing demand, employment choices, and economic status^26^. These factors have significant implications for farmers’ future residence preferences and consequently influence rural settlement patterns^27^. Therefore, studying the inherent connection between the family life cycle and housing choice can help us better comprehend and predict the specific housing needs of families at different stages. Farmers at different stages of the family life cycle may have diverse requirements and preferences for future residence. The family life cycle theory provides a crucial theoretical foundation for investigating farmers’ anticipated residential location preferences. Therefore, the objectives of this study are as follows: (1) To identify future residential site preferences from the perspective of the household life cycle. (2) To explore farmers’ perceptions regarding future residential selection and the factors influencing their choices. (3) To investigate the reasons behind farmers’ future residential location choices at different stages of the family life cycle and provide corresponding recommendations.

Although the existing literature has studied the influencing factors of farmers’ residential preferences, empirical research in China is relatively insufficient, especially from the perspective of the family life cycle. The uniqueness of the current study is to identify farmers’ future residential preferences from views of the individual farmer and family aspects. By exploring farmers’ residential preferences and the affecting factors, we provide a scientific basis for rural residential planning, social policy and rural development strategies. This study also provides a perspective for family-based decision-making studies from the family life cycle.

## Materials and methods

### Study area and sampled villages

As a prominent agricultural province and a key grain-producing region in China, Henan province has consistently held a leading position in terms of its planting area and yield for major grain crops like wheat. With a permanent population of 98.72 million as year of 2023 and notable population density^28^, Henan province exhibits distinctive regional characteristics and holds significant value on a national scale^29^. Furthermore, as China’s urbanization process accelerates, the number of floating populations in Henan province continues to grow, serving as a representative example that sheds light on rural social changes, the evolution of urban–rural relations, and the assessment of policy implementation effects in China. As the population density and number of floating populations increase in Henan province, substantial social and economic changes have occurred within rural areas. Notably, the prevalence of hollow villages and the rise in idle homesteads have emerged as prominent issues^30^. These not only pose significant challenges in the process of rural modernization in China but also serve as typical research cases for comprehending nationwide rural social and economic transformations.

**Figure 1:**
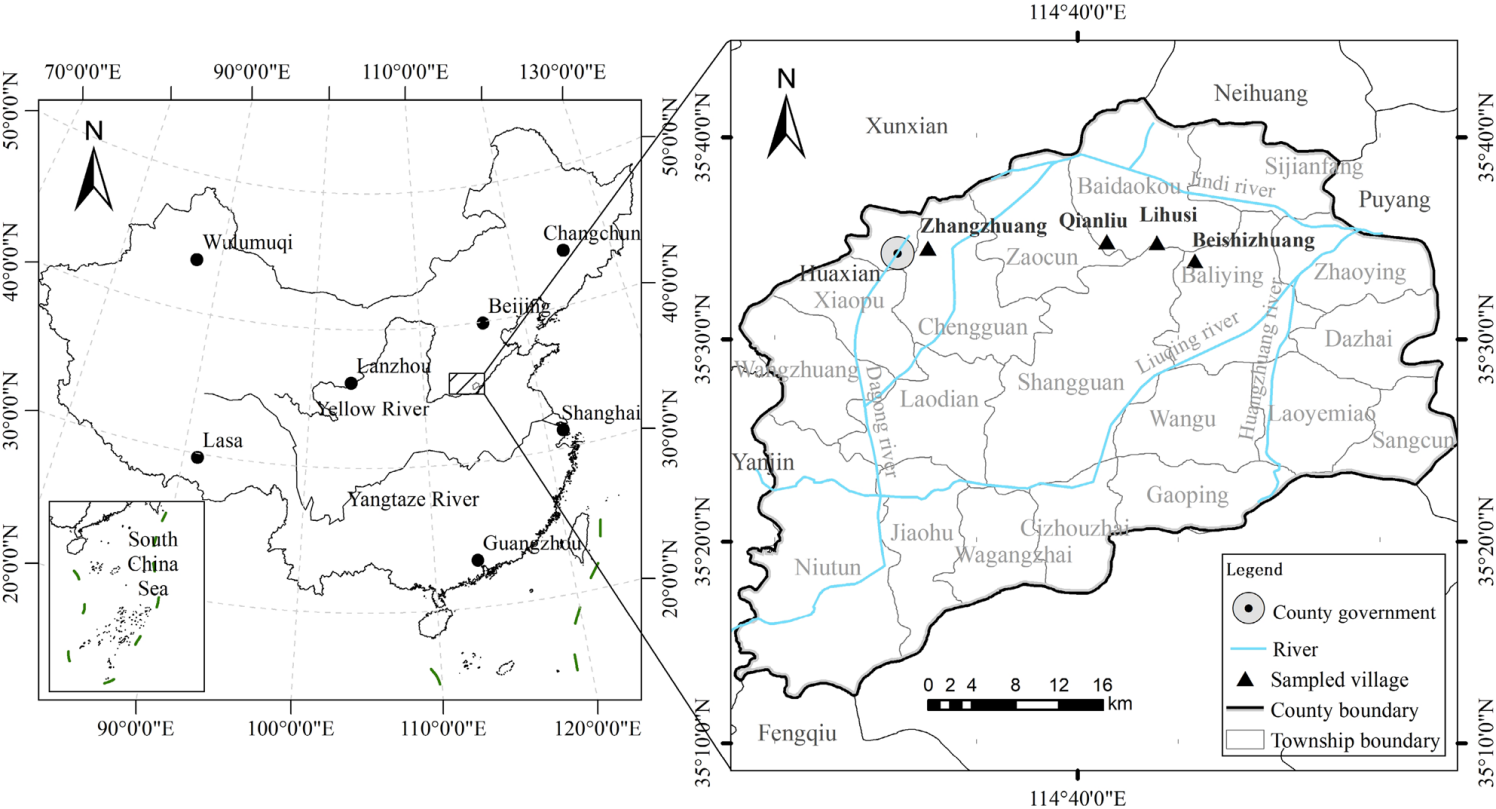
Location of the study area.

Huaxian, located in the northern part of Henan province (Fig. 1), boasting a predominantly flat terrain with an elevation of 60 m. The region experiences a humid climate, characterized by an annual precipitation range of 500–700 mm, while annual evaporation rates fluctuate between 1000 and 2000 mm. Covering a total area of 1814 km^2^, which includes 1340 km^2^ of arable land, Huaxian serves as the primary grain production zone within Henan province, focusing on the cultivation of wheat and corn as the main crops. The total population of Huaxian stands at 1,156,400, with 710,900 individuals residing in rural areas. The average annual household income in the region amounts to 11,087.81 USD, primarily derived from agriculture, animal husbandry, and labor export in rural areas.

For the purpose of this study, four villages were selected: Zhangzhuang, Qianliucun, Lihusi, and Beishizhuang. These villages were chosen based on their proximity to the county center and their sources of household income (Table 1).

**Table 1:** Distribution of sampled villages and households.

Town	Sampled village	Distance from county center/km	Sampled household	Percentage (%)	Main source of households income
Chengguan	Zhangzhuang	5.6	123	15.83	Handicraft, small business, and labor export
Baidaokou	Qianliucun	19.2	290	37.32	Agriculture and labor export
Baidaokou	Lihusi	24.1	257	33.08	Agriculture and handicrafts
Baliying	Beishizhuang	30.0	107	13.78	Agriculture and small business
Total	–	–	777	100	–

Zhangzhuang is positioned as a suburban village, located 5.6 km away from the county center. It is situated within the urban‒rural fringe of the town (Chengguan) and covers an area of 0.29 km^2^, including 0.18 km^2^ of arable land. This village is home to a total of 123 households, with an average annual household income of 12,510.60 USD. The primary sources of income in Zhangzhuang are the handicraft industry, small businesses, and labor export.

Qianliucun is classified as an outer suburb village and is situated 19.2 km away from the county center. It spans an area of 1.83 km^2^, with 1.58 km^2^ dedicated to arable land. The village accommodates a total of 290 households, with an annual household income of 11,821.07 USD. The primary sources of income in Qianliucun are agriculture and labor exports.

Lihusi is another outer suburb village, located 24.1 km away from the county center. The village covers an area of 0.16 km^2^, with 1.33 km^2^ allocated for arable land. Lihusi comprises 257 households, with an annual household income of 10,912.72 USD. The primary sources of income in Lihusi are agriculture and handicrafts.

Beishizhuang, which is also positioned as an outer suburb village, stands at a distance of 30.0 km from the county center. It covers an area of 0.70 km^2^, including 0.57 km^2^ of arable land, and is home to 107 households. The average annual household income in Beishizhuang is 10,340.61 USD (Table 4).

### Data collection

We conducted a questionnaire from July to September 2022 through face-to-face interviews. Prior to the interviews, households were notified by village leaders. Participation in the survey was voluntary. As a token of appreciation, each respondent received towels valued at USD 2 upon completion of the survey. The interviews were conducted with the head of the household, as they hold the decision-making power within the family. In cases where the wife’s preferred residence differed from that of the husband, the decision of the head of the household prevailed, as they typically have the highest decision-making authority regarding the choice of residence in the family. For adult children living with their parents, we investigated the head of the adult children’s household. We collected a total of 777 valid questionnaires, with the breakdown as follows: Zhangzhuang (123), Qianliucun (290), Lihusi (257), and Beishizhuang (107). Additionally, we conducted telephone interviews for unoccupied dwellings and obtained 93 responses in total: Zhangzhuang (15), Qianliucun (30), Lihusi (27), and Beishizhuang (21) (Table 1). The survey was administered by one postgraduate student and two senior college students serving as interviewers. The survey collected information the following aspects: (1) Characteristics of the household head (age, educational status, and health), living arrangements (solitary, couples residing together, two generations cohabiting, three generations living together). (2) Economic features of the households (annual household income, sources of income, level of economic growth, and land ownership). (3) Economic characteristics (annual income, sources of income, place of residence). (4) Attributes of the houses (affiliated properties, area, and year of construction) and future choice of residence (Table 2).

**Table 2:** Life cycle phasing of a family (Glick, 1947).

Life cycle stage of a family	Description	Identify
Formative stages	Initial households	Young newly married couple or may have children under the age of 16 and there are no individuals over the age of 65
Growth stages	Households in a stage of burden	The youngest child is under 16 years old, and the oldest member of the couple is over 65 years old
Maturity stages	Stabilized households	The youngest child in the family is over 16 years old, and there are no elderly individuals over 65 years old
Expansionist stages	Maintenance-stage households	The youngest child in the family is over 16 years old, and there are elderly individuals over 65 years old
Systolic stages	Empty-nesting households	After the division of family property, parents live alone

### Data analysis

Multiple unordered logistic regression models were used to analyse the dependent variable as an unordered multicategorical situation. The future residential sites for farm households include: father’s old house, current house, children's house in the village, house in the city (children’s house), and nursing home, which represent a multivariate unordered choice. In this study, we selected the multivariate unordered logistic model to analyze the factors influencing the residential space selection of farm households.

1 $$\ln \left[ {\frac{{P\left( {y = j\left| x \right.} \right)}}{{P\left( {y = J\left| x \right.} \right)}}} \right] = \alpha_{j} + \mathop \sum \limits_{k = 1}^{K} \beta_{jk} X_{k}$$

where P(y = j) denotes the probability of a farmer’s choice of the willingness, *X*_*k*_ denotes the kth independent variable affecting the future residential site selection, the independent variables are divided into three major categories: basic household characteristics, household economic characteristics and resource endowment, and *β*_*jk*_ denotes a vector of regression coefficients for the independent variables. Taking *J* as the reference type, the ratio $P\left( {y = j} \right)$/$P\left( {y = J} \right)$ of the probability of a farmer’s other types of residential site selection to the probability of a *J* type of residence, $\frac{{P\left( {y = \left. j \right|x} \right)}}{{P\left( {y = \left. J \right|x} \right)}}$ is the event-occurrence ratio, abbreviated odds.

This study aims to synthesize research findings from related fields both domestically and internationally. Foreign studies on farm households’ residential site selection have identified the main influencing factors as the family’s economic and social characteristics, housing construction, and the type of housing^31^. Domestically, research has shown that farmers’ willingness to select a residential location primarily takes into account social networks^32^, income, and family characteristics^33^. Based on these factors, this paper selects three types of influencing factors: personal characteristics, family characteristics, and resource endowment, as independent variables. As the empty nest period represents the final stage of the family life cycle, it is used as the reference group for this variable. The dependent variable in this study is the type of future residential site selection for farmers (ancestral house, current rural house, children’s house in the village, children’s house in the city, elder care facility). Given that elder care facilities are emerging as a new trend in China’s future old-age care, they are considered the reference group (Table 5).

### Division of family life cycle

The family life cycle represents the comprehensive trajectory of a family, including stages of formation, growth, maturation, and eventual decline or extinction^34^. This life cycle involves changes in family composition and membership, including the establishment and dissolution of marital unions, the birth and departure of children, and the passing of family members^35^. In 1947, Glick proposed a six-stage classification method to categorize surveyed families into different life cycle stages^22^. However, his structural division only considers the core and stable forms of the family, assumes a family size of two, and overlooks other forms such as single-child families, unmarried individuals, divorced individuals, and remarried individuals, resulting in theoretical limitations. Therefore, when applying the family life cycle theory to study various regions, it is essential to consider China’s national conditions and regional differences^36^. In rural China, the birth of a family is not marked by marriage but by the division of the family into separate households. This leads to a more complex family structure, with some elderly individuals living with their children^37^. As a result, the family life cycle theory acknowledges that families have different contents and tasks in different life courses but does not provide a defined set of stages. Researchers can thus divide the stages of the family life cycle based on the specific research content and purpose, aligning them with the research theme. Taking into account the structure of rural households in China and local realities, and considering the focus of our study, the classification is based on the life cycle of households with adult children, and we classify families into five distinct life cycle stages: the initial stage, burden stage, stable stage, maintenance stage, and empty nest stage (Table 2). This classification is grounded in the consideration of whether teenagers have reached the age of 16 and whether elderly individuals have attained the age of 65, serving as the basis for categorizing the family life cycle. Given the context of rural China, where multiple generations often live together, we did not account for the core family structure of couples. Thus, in this investigation, we relied on the family life cycle division proposed by scholars Wang Wei and Wu Haitao, combined with our field research, we classified families into five distinct life cycle stages, namely, the initial stage, burden stage, stable stage, maintenance stage, and empty nest stage (Table 2).

(1)The initial stage of a family pertains to couples who have recently married. Within this stage, family members may not yet have children or may have children under the age of 16. Additionally, there are no individuals over the age of 65. (2) The burden stage includes couples with one or more young children, where the youngest child is under 16 years old, and the oldest member of the couple is over 65 years old. (3) The stabilization stage corresponds to a phase in which children gradually mature and enter adolescence. In this stage, the youngest child in the family is over 16 years old, and there are no elderly individuals over 65 years old. (4) The maintenance stage occurs when a child leaves home to embark on adult life. At this point, the youngest child in the family is over 16 years old, and there are elderly individuals over 65 years old. (5) The empty nest period refers to the establishment of an independent living arrangement after the children leave the family, following the separation of the parents, the division of family property, and when only the parents live alone (Table 2).

### Ethical approval

This research study is performed per the principles of the Declaration of Helsinki. It did not require ethical approval because the participants willingly shared their future residential preference. The data for this study were obtained through a voluntary survey involving adult participants. Throughout the course of this research, we adhered to relevant ethical guidelines, ensuring the respect of participants' rights and privacy. The design and implementation of the survey followed ethical principles, including the protection of participant privacy, assurance of data confidentiality, and respect for voluntary participation. We further confirm that this study posed no risks of physical, psychological, or social harm to participants, and all research procedures complied with international ethical research standards. We declare that all data collection and questionnaires survey of the manuscript is under supervised and approved by Academic and Review Board of Henan Polytechnic University.

### Informed consent

The authoritative office gave written consent, and the authors got everyone’s verbal informed consent.

## Result

### Characteristics of sampled households

**Figure 2:**
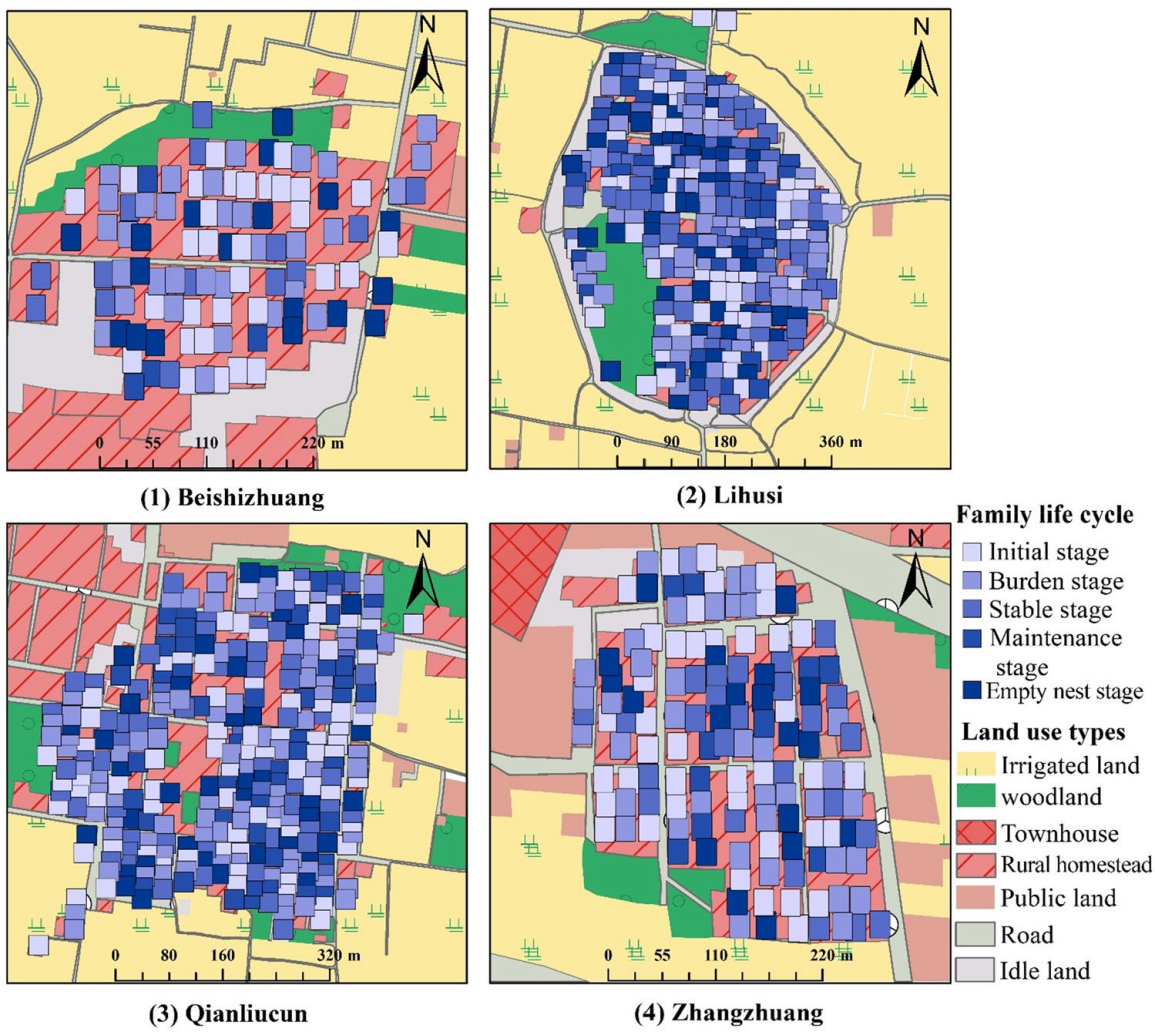
Life cycle stage distributions of families in different types of villages.

The mean age of household heads stands at 51.40 (± 13.51) years, while the average years of education received is 7.68 (± 3.55) years. The average annual household income is 11.08 (± 20.96) thousand USD (Table 3), and the average number of children per household is 2.15 (Table 5). On average, each family possesses 1.37 (± 0.92) houses, and approximately 32.56% of families own two or more houses. Among the four sampled villages, Beishizhuang boasts the highest average number of houses per household, with an average of 1.73 (± 2.05) houses per household, followed by Lihusi (1.44 ± 0.56), Zhangzhuang (1.38 ± 0.54), and Qianliucun (1.16 ± 0.45) (Table 3). Regarding the distribution of life cycle stages across the villages, the burden stage holds the largest proportion, spanning from 26.17 to 30.35%. It is succeeded by the initial stage, which ranges from 23.79 to 30.89%. The stable stage ranges from 12.15 to 21.03%, while the empty-nest stage varies from 12.41 to 23.36%. The maintenance stage constituted 11.71% of the distribution (Table 4 and Fig. 2).

**Table 3:** Characteristics of sampled household (mean ± SD).

Villages	Age of household head (years)	Education of household head (years)	Number of homesteads owned	Household annual income (1000 USD)	Family life cycle stages
Zhangzhuang	54.28 ± 14.44^a^	7.85 ± 3.54	1.38 ± 0.54^bc^	12.51 ± 23.86	1.99 ± 1.19
Qianliucun	49.80 ± 13.32^b^	7.84 ± 3.37	1.16 ± 0.45^c^	11.82 ± 22.04	2.47 ± 1.10
Lihusi	52.23 ± 13.11^ab^	7.46 ± 3.60	1.44 ± 0.56^b^	10.91 ± 19.68	2.43 ± 1.17
Beishizhuang	50.65 ± 13.13^ab^	7.55 ± 3.87	1.73 ± 2.05^a^	10.35 ± 15.86	2.79 ± 1.36
Mean	51.40 ± 13.51	7.68 ± 3.55	1.37 ± 0.92	11.08 ± 20.96	2.45 ± 1.16

Note: Family life cycle stages: 1, initial stage; 2, burden stage; 3, stable stage; 4, maintenance stage; 5, empty nest stage. Different letters within a column indicate statistically differences at *p* < 0.05, Tukey’s post-hoc tests: (a > b > c). “a”, “b” and “c” represent different groups of means, and the alphabetical order reflects the ranking of the means from highest to lowest, “ab”, “bc” and “ac” indicated that the difference between the corresponding groups did not reach a significant level (*p* ≥ 0.05).

**Table 4:** Family life cycle and vacant housing ratio.

Village	Initial stage			Burden stage			Stable stage			Maintenance stage			Empty nest stage		
	Number of household	Proportion (%)	Vacant house (%)	Number of household	Proportions (%)	Vacant house (%)	Number of household	Proportions (%)	Vacant house (%)	Number of household	Proportions (%)	Vacant house (%)	Number of household	Proportions (%)	Vacant house (%)
Beishizhuang	30	28.04	16.67	28	26.17	10.71	13	12.15	7.69	11	10.28	0	25	23.36	4
Lihusi	66	25.68	1.51	78	30.35	5.13	45	17.51	15.56	30	11.67	16.67	38	14.79	10.53
Qianliucun	69	23.79	4.35	83	28.62	7.23	61	21.03	9.84	41	14.14	4.88	36	12.41	5.56
Zhangzhaung	38	30.89	5.23	36	29.26	2.78	22	17.89	13.64	9	7.32	33.33	18	14.63	11.11
Total	203	26.13	5.42	225	28.96	6.22	141	18.15	12.06	91	11.71	10.99	117	15.06	6.84

Note: Vacant house excludes housing that is occupied more than two months per year.

### Residents’ preference for housing choices in the future

**Figure 3:**
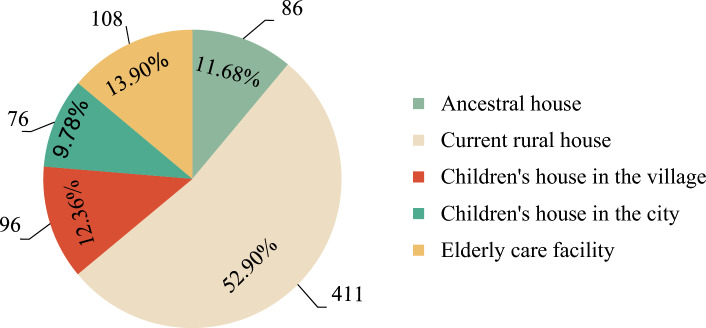
Proportion of future residential preference.

The primary preference for future housing selection among residents is residing in their current rural house, accounting for 52.90% of the selections. This is followed by a preference for elderly care facilities (13.90%), children's houses within the village (12.36%), and ancestral houses (11.68%). The option with the lowest ratio is residing in children’s houses in the city (9.78%) (Fig. 3).

**Figure 4:**
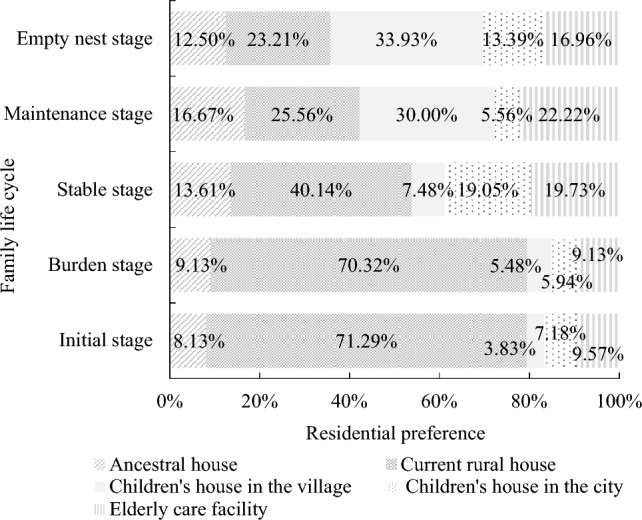
Proportions of future residential preference for different types of households.

**Figure 5:**
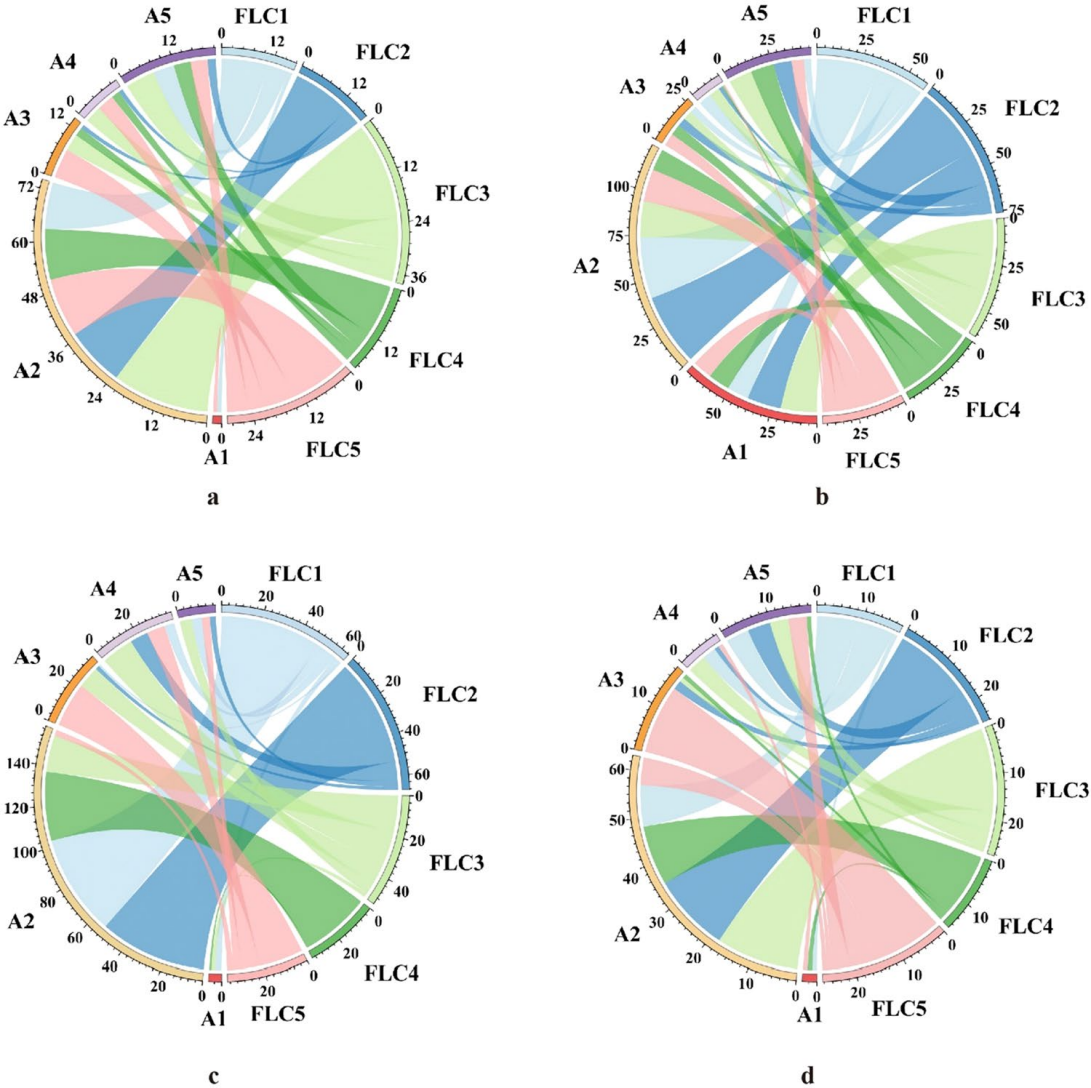
Chord diagram of future residential preference for different types of family. Notes: The flow from FLC to A represents the choice of residential location during different stages of the lifecycle, and the area represents the proportion of each choice. A1: Ancestral house, A2: Current rural house, A3: Children’s house in the village, A4: Children’s house in the city, A5: Elderly care facility. FLC1: Initial stage, FLC2: Burden stage, FLC3: Stable stage, FLC4: Support stage, FLC5: Empty nest stage. Distance from village to county center: d > c > b > a.

Residents’ intentions regarding their future living place exhibit variations across different stages of the family life cycle. Individuals in the initial stage, burden stage, and stable stage of the family life cycle tend to favor their current rural house as their future abode, with proportions ranging from 40.14 to 71.29%. Conversely, for families in the maintenance stage and empty-nest stage, living in their children’s village house become the preferred choices, with ratios ranging from 30.00 to 33.93%. Notably, the proportion of residents selecting elderly care facilities as their future housing settlement in the stable stage, maintenance stage, and empty-nest stage is higher, ranging from 16.96 to 22.22%, compared to the initial stage and burden stage, which range from 9.13 to 9.57% (Figs. 4 and 5).

Furthermore, the proximity of villages to the county center influences residents’ intentions for future housing settlement. Housing choices differ among villages, with those closer to the county center showing a tendency for fewer residents to select living in their children’s houses in the future and a greater inclination toward elderly care facilities as their future living place (Table 6).

### Factors affecting residents’ future residential preference

We conducted a logistic regression analysis to investigate the effect of family life cycle, individual characteristics, socioeconomic factors and resource endowment on the likelihood of future residential preference. A total of 11 variables were included in the model and elderly care facility set as a reference variable of the model (Tables 5, 6 and 7).

**Table 5:** Measures and description of future residential preference and its influencing factors.

Variables name	Code	Items	Mean	SD
Dependent variable				
Future residential preference	FRP	Ancestral house = 1, current rural house = 2, children’s house in the village = 3, children’s house in the city = 4, elderly care facility = 5	2.60	1.24
Independent variables				
Family life cycle	FLC	Initial households = 1, burden stage = 2, stabilized households = 3, maintenance-stage households = 4, empty-nesting households = 5	–	–
Personal characteristic of household head				
Household head Age	HHA	Years	51.44	13.51
Health	HEAL	Very poor or poor = 1, fair = 2, good = 3, very good = 4	2.30	0.96
Education	EDU	Years	7.68	3.55
Family characteristics				
Household annual income	HAI	Unit: 1000 USD	11.08	20.96
Degree of household economic growth (latest 5 years)	DEG	Rapid or slow decrease = 1, constant = 2, slow growth = 3, rapid growth	–	–
Living pattern	LP	Solitary = 1, couples living together = 2, two generations living together = 3, three generations live together = 4	–	–
Number of children	NC	–	2.15	0.79
Endowment of resources				
Village location	VL	Distance to county center: 1–10 km = 1; 11–20 km = 2; 21 km -30 km = 3; 30km-40km = 4	2.55	0.92
Number of homesteads owned	NH	–	1.37	0.92
Acres of land owned	AL	–	5.92	9.18

**Table 6:** Influence of family life cycle on the willingness of residents to locate their dwellings.

Independent variable	Ancestral house			Current rural house			Children’s house in the village			Children’s house in the city			Collinearity test	
	B	Significance	OR	B	Significance	OR	B	Significance	OR	B	Significance	OR	Tolerance	VIF
Initial stage	0.30	0.74	1.35	4.42**	0.00	83.34	1.02	0.26	2.76	− 1.37	0.23	0.25	0.71	1.41
Burden stage	0.32	0.68	1.38	3.79**	0.00	44.40	0.72	0.38	2.06	− 1.01	0.29	0.36		
Stable stage	− 0.48	0.47	0.62	1.66**	0.01	5.23	0.70	0.33	2.02	0.19	0.80	1.21		
Maintenance stage	− 0.38	0.62	0.69	2.22**	0.00	9.21	2.88**	0.00	17.80	− 1.57	0.11	0.21		
Empty-nest stage	Reference													
HHA	0.02	0.49	1.02	0.08**	0.00	1.08	0.10**	0.00	1.10	− 0.06	0.11	0.94	0.57	1.76
HEAL (very poor and poor)	−0.17	0.86	0.84	0.34	0.64	1.40	-0.89	0.35	0.41	− 1.64	0.13	0.19	0.44	2.27
HEAL (general)	0.31	0.68	1.36	− 0.07	0.90	0.93	− 1.93*	0.03	0.15	− 1.54	0.08	0.22		
HEAL (good)	0.22	0.73	1.24	− 0.24	0.63	0.79	− 1.19	0.10	0.31	− 1.67*	0.03	0.19		
HEAL (very good)	Reference													
EDU	0.01	0.93	1.01	− 0.06	0.24	0.94	− 0.09	0.22	0.92	0.01	0.90	1.01	0.57	1.75
HAI	− 0.20	0.15	0.98	− 0.01	0.41	0.99	− 0.01	0.33	0.99	− 0.11*	0.01	0.90	0.87	1.15
DEG (rapid or slow decrease)	− 0.41	0.64	0.66	0.36	0.25	1.43	1.94*	0.04	6.96	− 0.43	0.69	0.65	0.57	1.76
DEG (constant)	− 0.29	0.63	0.74	0.03	0.95	1.03	1.35	0.08	3.87	− 1.20	0.15	0.30		
DEG (slow growth)	0.01	0.98	1.01	0.33	0.48	1.39	1.09	0.12	2.96	− 0.84	0.23	0.43		
DEG (rapid growth)	Reference													
LP (solitary)	− 0.75	0.16	0.47	1.83**	0.00	6.23	17.69**	0.00	1.02	− 5.16	0.12	1.33	0.90	1.11
LP (two-generation cohabiting)	− 1.35**	0.01	0.26	1.72**	0.00	5.60	10.26*	0.01	1.48	− 3.25**	0.00	0.04		
LP (three-generation cohabiting)	Reference													
NC	− 0.01	0.95	0.99	− 0.04	0.82	0.96	0.45	0.54	1.16	0.04	0.88	1.04	0.96	1.05
VL (1 -10 km)	1.33	0.17	3.79	− 0.94**	0.00	0.39	− 0.13	0.86	0.88	1.06	0.16	2.90	0.85	1.18
VL (11–20 km)	1.99*	0.03	7.31	0.61	0.20	1.83	0.09	0.89	1.09	2.61**	0.00	13.60		
VL (20km-30km)	3.70**	0.00	40.55	− 0.18	0.51	0.83	0.25	0.70	1.28	0.11	0.84	1.12		
VL (30km-40km )	Reference													
NH	0.09	0.73	1.10	0.04	0.89	1.04	− 0.06	0.87	0.94	0.75*	0.04	2.11	0.95	1.06
AL	− 0.16**	0.00	0.85	− 0.01	0.96	0.99	− 0.07	0.00	0.93	− 0.03	0.48	0.97	0.87	1.15
Nagelkerke Pseudo R-squared	0.76													
F	9.57													
Sig	0.00													

Note: The elderly care facility is the reference item of the model. Reference means that other variables in this category refer to this variable. OR: Odd Ratio. * Significant at* p* < 0.05. ** Significant at *p* < 0.01.

HHA, Household head Age; HEAL, Health; EDU, Education; HAI, Household annual income; DEG, Degree of household economic growth (latest 5 years); LP, Living pattern; NC, Number of children; VL, Village location; NH, Number of homesteads owned; AL, Acres of land owned.

**Table 7:** Likelihood ratio test.

Effect	The model fitting conditions simplify the-2 log-likelihood of the model	Likelihood ratio test		
		Chi-square	Degrees of Freedom	Significance
Education	1121.947	2.568	4	0.63
Acres of land owned	1140.287	20.909	4	0
Number of children	1120.375	0.997	4	0.91
Number of homesteads owned	1124.249	4.87	4	0.3
Household annual income	1131.55	12.172	4	0.02
Household head Age	1142.885	23.506	4	0
Family life cycle	1290.708	171.329	16	0
Health	1137.433	18.054	12	0.114
Living pattern	1600.467	481.088	12	0
Degree of economic growth	1130.428	11.05	12	0.525
Village location	1242.938	123.559	12	0

Note: Square statistics is the difference between the − 2 log-likelihood of the final model and the simplified model. The simplified model is formed by omitting an effect in the final model. The null hypothesis is that all the parameters of this effect are 0.

Compared with living in elderly care facility, families in the initial stage (odds ratio, OR = 83.34,* p* < 0.01), burden stage (OR = 44.40,* p* < 0.01) and stable stage (OR = 5.23, *p* < 0.05) are more willing to choose to live in the current rural house in the future. Families in the maintenance stage (i.e., supporting families) are more likely to choose to live in a current rural house (OR = 9.21, *p* < 0.01) and their children’s houses in villages (OR = 17.80, *p* < 0.01) than live in elderly care facilities.

Elders are likely to choose to live in a current rural house (OR = 1.08, *p* < 0.01) and children’s houses in villages (OR = 1.10, *p* < 0.01). Households with high annual incomes are reluctant to choose to live in their children’s houses in cities (OR = 0.90,* p* < 0.05) in the future. By contrast, farmers who perceived low household income growth tend to live in their children’s houses in villages (OR = 6.96, *p* < 0.05) in the future. Farmers with low health condition were less likely to choose to live in their children’s house in the villages (OR = 0.15, *p* < 0.05) and cities (OR = 0.19, *p* < 0.05) in the future compare to elderly care facilities.

Families living alone and two-generation families, compared to those who live in elderly care facilities, choose to continue living in their current rural houses (OR = 6.23 and 5.60, *p* < 0.01) and children’s houses in villages (OR = 1.02, *p* < 0.01 and OR = 1.48, *p* < 0.05). By contrast, two-generation families are less likely to choose to live in ancestral houses (OR = 0.26, *p* < 0.05) or children’s houses in cities (OR = 0.04, *p* < 0.01). Households close to county centres are unwilling choose to live in their current rural houses (OR = 0.39, *p* < 0.01) and prefer to live in their children’s houses in cities (OR = 13.60, *p* < 0.01) as their future residential sites. Farmers with more houses are willing to live in their urban children’s houses (OR = 2.11, *p* < 0.05) in the future. By contrast, farmers owning more farmland are less willing to stay in their ancestral houses (OR = 0.85, *p* < 0.01) (Table 6).

## Discussion

Farmers’ attitudes regarding their future residential preference play a crucial role in rural planning and the effective management of hollow villages^38^. In China, residential locations hold particular significance for farmers due to their connection with land, traditional culture, and rural well-being^39^. Attitudes toward future residential preference among farmers are complex and divergent across different stages of the family life cycle, serving as significant barriers to the sustainable development of rural areas. In this study, we selected villages with similar family structures but varying geographical locations, income sources, and livelihoods, distinct from the main grain-producing zones of China. We found that the majority of households express a willingness to choose their current rural house as their future residence. This choice is significantly influenced by the characteristics of household heads, family income and livelihood, affiliated houses and lands, and the life-cycle stages of households.

The preference and choice of farmers’ future residence can be influenced by the family life cycle. Based on our research findings, it is evident that the family life cycle plays a significant role in determining both the future ancestral house and the current house choices. Our research indicates that households in the burden stage are more likely to make these choices, and there is a clear correlation between the family life cycle and socio-economic phenomena such as non-agricultural labor migration^40^, abandonment of rural homesteads, and family migration. This finding is in line with the research conducted by Xia et al.^41^.

Regardless of the stage in the family life cycle, our study finds that farmers are generally more inclined to choose to stay in existing homes than in elderly care facilities. Except for households in the maintenance stage and empty nest stage, who prefer living in their children’s homes within the village, households in all five stages primarily choose to continue residing in their current rural houses in the future. The family life cycle reflects the diverse ages and compositions within a family, and how it evolves over time. In smaller households, middle-aged individuals often experience the pressure of caring for elderly family members. Consequently, they tend to engage in livelihood activities outside their hometowns during the maintenance stage. As the family stages evolve, housing choices also change^29^.

China has a rural population of approximately 500 million individuals^42,43^. Zhong^44^ emphasised the importance of gradually reducing the disparities between urban and rural development and enhancing the living standards of the rural population. In urbanization, the sustainable development of rural areas must be prioritised.

Traditional village culture and customs have the potential to influence the future residential preferences of farming households^45^. Traditional rural areas are characterized by subsistence agricultural production, with one’s hometown representing traditional Chinese agricultural civilization and serving as a significant psychological and behavioral characteristic among traditional farmers^46^. Chinese households adhere to traditional living patterns, leisure activities, social interactions, and housing styles. Multigenerational cohabitation is prevalent among the majority of households^47^. The strong emotional attachment of Chinese individuals to their homes and hometowns influences their decision to return to their hometowns in old age. At present, farmers’ way of life has an impact on their future choices of residence^48^. Research by Dueppen highlights that in ancient West Africa, farm dwellings served various purposes, such as farming, cultivation, and habitation, with multigenerational cohabitation being the dominant settlement pattern of that era^49^. Long-term living arrangements, such as living alone or with two or three generations, significantly affect farmers’ future housing choices^50^. Elderly individuals who live alone are inclined to either stay in their current rural house or relocate to their children's homes in rural areas. Their decision is primarily influenced by their familiarity with the surroundings and the absence of consistent company from their children over extended periods^51^. Therefore, an empirical examination of the future residential preferences of rural households in traditional Chinese rural areas, from the perspective of the family life cycle, can contribute to the enhancement of rural development and serve as a reference for future rural planning.

The role of the household head in making final decisions regarding family matters, including housing choices and land management, is of crucial importance. Various factors related to the household head, such as age, health, and education, can have an impact on their future residential preferences. Our study indicates that older household heads often face difficulties in adapting to urban life and generally prefer remaining in their rural homes in the future. This finding is consistent with a report from New Zealand which suggests that older individuals tend to prefer familiar areas^52^. On the other hand, young people in developing countries tend to migrate frequently from rural to urban areas due to the need for urban employment to sustain their livelihoods^53^. Household heads in poor health are likely to rely on the companionship and care of their children, leading them to choose to live with their children in the future. This is supported by research conducted by Ranga^54^. In many developing countries, the aged agricultural workforce and the migration of young people away from agriculture are common trends^55^.

The financial situation and livelihood of a family are crucial factors that significantly influence the quality of family life and future residential preferences. Family income is also a determining factor in rural development^56,57^. Households with lower incomes are less likely to afford homes in urban areas and are generally markedly inclined to remain in rural settings. Conversely, households with higher incomes have the means and the desire for improved residential facilities and medical services, making them more likely to opt for elderly care facilities in the future. The livelihoods of the sampled households include various activities such as agriculture, labor, handicrafts, and small businesses, with agriculture often serving as the primary source of livelihood. Therefore, most households are disinclined to move to urban areas. This aligns with traditional rural settings in countries like India, where the primary sources of income are land and agriculture and higher income levels lead to better housing conditions^58^. Increased agricultural productivity, as demonstrated by Adetoro^59^, can raise income, enhance livelihoods, improve household consumption levels, and influence housing choices among households.

Affiliated properties and lands hold significant importance for a household’s sustenance and play a pivotal role in determining whether households choose to remain in the village in the long term. Some households not only own homes within the village, but also invest in commercial properties in the county or city. These additional properties are often used for purposes such as marriages or providing educational opportunities for their children^60^. Research has shown that households with multiple properties are more likely to pursue urban residences in the future. Furthermore, land ownership has a significant impact on rural economic development^61^. In regions like Amhara, land ownership is considered a criterion for socioeconomic development, and possessing more land gives households greater flexibility in terms of housing choices. Our research findings align with this perspective, indicating that farmers with larger land holdings are less inclined to choose ancestral houses as their future residences. A similar trend has been observed in Kenya^62^ and Vietnam^63^, where traditional households heavily reliant on agriculture base their future housing decisions on the extent of their land ownership. Balezentis^64^ has highlighted that the closer a village is to a city, the lower the participation of young individuals in agriculture. This emphasizes the importance of convenient transportation and diverse agricultural opportunities in attracting young migrant workers to return to their villages in old age. The housing environment plays a crucial role in the decision-making process of migrant workers looking to return^52^. Consequently, our study underscores the necessity of gradually improving the management of rural homesteads and implementing better land use planning.

Furthermore, our study raises new questions regarding the aging population in rural areas within the context of rapid urbanization. In China, the migration of a significant number of young rural laborers to urban areas has resulted in reduced agricultural productivity in villages. This, in turn, has led to an increase in the rural elderly population. The implications of this phenomenon extend not only to China but also to other countries with predominantly smallholder agricultural systems^65^. Afghanistan, for example, where over 54.5% of the population lives in poverty and agriculture is the primary livelihood in rural areas, relies heavily on arable land and cultivation for the survival of its farmers^66^. Kenya^62^ and Vietnam^63^ face a similar situation, with traditional households heavily dependent on agriculture. The extent of land ownership significantly influences their future housing choices. Therefore, the introduction of new models of agricultural production becomes crucial in order to incentivize young laborers to remain in rural areas. Simultaneously, improving rural infrastructure and enhancing household satisfaction are necessary measures to mitigate the phenomenon of village hollowing out^65,67^.

As farmers age and young individuals with higher levels of education show reluctance to engage in agricultural activities, there is a growing inclination among young people to live in urban areas in the future^68^. Our study also reveals that healthier household heads exhibit reduced dependence on their children, making them less likely to choose to live with their children in the future and instead opt for elderly care facilities. Therefore, focusing on the health of the elderly residing in rural areas and enhancing the provision of medical and healthcare services are of utmost importance. Though education itself may not directly determine future residential preferences, the low education levels among elderly individuals in rural areas are cause for concern. Efforts should be made to improve the education level of farmers in the future. This can be achieved through the establishment of farmer-focused schools, increased investments in rural educational resources, and the development of enterprises to provide additional income opportunities for rural residents. These strategies increase the likelihood that farmers will choose to stay in the countryside and minimize the abandonment of unused homesteads.

## Conclusion

This study examines farmers’ future residential preferences and the affecting factors from the family life cycle perspective in rural China. Increased income, access to education for children, healthcare availability, and transportation options are the primary drivers behind their decision to leave their villages. Meanwhile, rapid urbanization has resulted in a rise in empty nesters and unused properties in rural areas, exacerbating the issue of rural depopulation. Our findings indicate that the majority of households choose to stay in their current rural residences in the future, while an increasing number of younger individuals are relocating to urban centers, contributing to a decline in rural productivity. The age and health status of the household head, household income and sources of livelihood, property ownership, and current stage of residence are potential factors that influence their future living preferences. Based on these findings, interventions aimed at promoting the efficient utilization of unused rural properties should be contemplated: (1) Improving rural medical services and infrastructure is essential to address the healthcare needs of the elderly population in rural areas. (2) Establishing farmers’ schools is crucial for enhancing the education levels of farmers, particularly elderly individuals, to help them adapt to rural development. (3) Enhancing employment opportunities and security for rural–urban migrants can attract laborers back to their villages, mitigating the issue of rural depopulation.

## Data Availability

The data presented in this study are available on request from the corresponding author.
